# Surface Characteristics and Hydrolytic Stability in Milled and 3D-Printed PMMA Dental Materials

**DOI:** 10.3390/polym18050597

**Published:** 2026-02-28

**Authors:** Liliana Porojan, Flavia Roxana Bejan, Roxana Diana Vasiliu, Mihaela Ionela Gherban, Lavinia Cristina Moleriu, Anamaria Matichescu

**Affiliations:** 1 Department of Dental Prostheses Technology (Dental Technology), Center for Advanced Technologies in Dental Prosthodontics, Faculty of Dental Medicine, “Victor Babeș” University of Medicine and Pharmacy Timișoara, Eftimie Murgu Sq. No. 2, 300041 Timișoara, Romania; sliliana@umft.ro (L.P.); roxana.vasiliu@umft.ro (R.D.V.); 2 National Institute for Research and Development in Electrochemistry and Condensed Matter, 300569 Timisoara, Romania; mihaelabirdeanu@gmail.com; 3 Department of Functional Science, Medical Informatics and Biostatistics, “Victor Babeș” University of Medicine and Pharmacy Timișoara, Eftimie Murgu Sq. No. 2, 300041 Timisoara, Romania; 4 Department of Preventive, Community Dentistry and Oral Health, Center for Advanced Technologies in Dental Prosthodontics, Faculty of Dental Medicine, “Victor Babeș” University of Medicine and Pharmacy Timișoara, Eftimie Murgu Sq. No. 2, 300041 Timișoara, Romania; matichescu.anamaria@umft.ro

**Keywords:** dental PMMA, 3D printing, CAD/CAM milling, water sorption, surface roughness, topographic characteristics, hydrolytic stability

## Abstract

This study investigated how fabrication method (milling versus 3D printing) affects the water sorption and solubility of PMMA dental materials, and how surface characteristics affect hydrolytic stability. Fifty-six PMMA samples were divided into three groups fabricated from CAD/CAM milled discs (Group A: I–III) and four groups from 3D-printed resin (Group B: IV–VII), each subjected to distinct postprocessing protocols. Water sorption (wsp) and solubility (wsl) were measured after immersion in distilled water at 37 °C for 24, 48, and 72 h, and 7 and 14 days. Surface topography and nanoroughness were assessed using atomic force microscopy (AFM). Statistical descriptive analyses were followed by correlation analyses. Milled PMMA demonstrated significantly lower water sorption and negative solubility (mass loss), indicating material dissolution. In contrast, 3D-printed PMMA showed higher water sorption and positive solubility (mass gain), reflecting water incorporation and polymer swelling. The kinetic profiles differed: milled PMMA displayed a monophasic absorption curve, while 3D-printed PMMA exhibited a biphasic pattern with accelerated water uptake after 72 h. AFM analysis revealed that 3D-printed surfaces had significantly greater nanoroughness than milled surfaces. Strong positive correlations were observed between surface roughness parameters (Sa, Sy) and water sorption capacity. The fabrication method was found to influence the hydrolytic stability of PMMA dental materials. Milled PMMA demonstrated superior stability, with lower water uptake, smoother surfaces, and lower leaching solubility. In contrast, 3D-printed PMMA exhibited increased surface roughness and water sorption, attributed to its layered microstructure and nanoporosity. Surface topography emerged as a strong predictor of wsl, related to hydrolytic degradation. For clinical applications, milled PMMA is recommended for long-term use requiring durability, whereas 3D-printed PMMA may be appropriate for short-term applications with optimised postprocessing.

## 1. Introduction

The transition from conventional laboratory techniques to digital workflows has a significant impact on all aspects of restorative dentistry. Initial digitalisation occurred through computer-aided design and computer-aided manufacturing (CAD/CAM) systems, which utilise subtractive milling of prefabricated blocks. However, these methods are inherently limited by material waste, bur wear, and constraints in producing highly complex geometries. In contrast, additive manufacturing (AM), particularly three-dimensional (3D) printing, constructs restorations layer by layer from virtual designs. These were introduced to address some disadvantages of subtractive systems, enabling finer details (e.g., improved undercuts and morphology). They are considered fast and economical techniques for the precise manufacturing of models and various types of prostheses with minimal material waste [1,2,3,4,5,6]. However, there is limited knowledge regarding the comparison of CAD/CAM-milled restoration and three-dimensional-printed restoration with respect to material properties, the effects of manufacturing techniques, and long-term behaviour.

Polymethyl methacrylate (PMMA) is frequently the basis for digital processing because of its high stability and endurance [6]. In contrast to 3D-printed PMMA resins, which have low polymerisation rates that result in poor surface integrity, CAD/CAM-milled PMMA resins are dense due to their high polymerisation rate, industrial manufacture, and high crosslinking. Research revealed that whereas HDMA (hexamethylene glycol dimethacrylate), the monomer utilised in light-polymerised resins, is hydrophilic, CAD/CAM-milled and traditionally processed PMMA resins contain hydrophobic MMA (methylmethacrylate)-based monomers [7]. Fillers, on the one hand, enhance the mechanical properties of printable materials; on the other hand, they increase the risk of air entrapment, increase anisotropy, and may reduce accuracy if dispersion is unequal [8].

PMMA has become the preferred material. Its qualities, including ease of processing, high optical properties, durability, wear resistance, and high stability, are the reason for this favour. These characteristics may experience negative changes once the restorations are exposed to the intraoral dampness environment. Postprocessing is required to mitigate these disadvantages [5,9].

Key characteristics of 3D-printed restorations, including mechanical properties, biocompatibility, durability, dimensional accuracy, and aesthetic appeal, are significantly affected by a number of molecular changes that occur during postprocessing. This phenomenon, based on digital accuracy and material science, is not just a technical achievement [5,6,10]. Cleaning, curing, and finishing are important processes that affect the finished product’s mechanical strength, appearance, and biocompatibility [8,11].

To achieve the required mechanical and biological performance, three-dimensional-printed dental resins require a two-stage postprocessing strategy that includes solvent washing to remove uncured resin and thermal and UV post-curing. To retain the viscosity required for layerwise synthesis, these materials typically comprise (di)methacrylate monomers and a relatively low inorganic filler fraction. This naturally restricts the degree of conversion and leaves residual monomers in the organic matrix. To remove adhering uncured resin, printed objects are frequently cleaned with alcohol-based solvents (e.g., isopropyl alcohol and tripropylene glycol monomethyl ether). Insufficient washing may allow surface remnants to polymerise in situ, compromising dimensional accuracy, marginal fit, and possibly increasing intraoral release of residual monomer. Long-term washing can minimise residual monomer content, but too much alcohol exposure might break down the polymer network, resulting in decreased mechanical properties because of partial dissolution and irreversible plasticisation of the resin matrix [1,5,12,13,14]. Long-term chemical washing, however, weakens the polymer network and permits solvent penetration from the surface into the resin matrix, increasing water sorption and solubility, decreasing flexural strength, and producing serious surface defects. Inorganic fillers can also be leached by excessive post-washing, resulting in gaps beneath the surface that increase solvent absorption and lower polymer density, deteriorating the material’s chemical and physical qualities [15].

Besides the duration, selecting the right solvent for the washing process must be associated with fine-tuning post-cure device settings like exposure time, temperature, and wavelength, which together result in noticeable improvements in the finished product [5]. For 3D-printed outputs, the degree of curing is typically only between 60% and 70%. Thus, strength and durability can be greatly improved by increasing the degree of curing. To improve the postcuring process, numerous postcuring methods and products have been developed [16]. Controlling oxygen inhibition during postpolymerisation enhances the degree of conversion of 3D-printed PMMA resins, thereby improving their physical-mechanical properties and surface characteristics and suggesting it as a promising clinical polymerisation strategy [17].

A significant factor impacted by the postprocessing process is water absorption, which has a detrimental effect on the biocompatibility and rate of degradation of the dental 3D-printed parts in addition to other negative effects like dimensional changes, retention loss, and margin contour fracture of the dental crowns. In resin materials, water is usually absorbed, allowing it to enter voids, such as micro-voids. The strength of materials is diminished, chemical degradation occurs, and leftover monomers are released as a result of these water-polymer chain interactions. Because 3D-printed parts are subjected to higher temperatures for shorter periods than traditional heat-cured materials, they often exhibit higher water solubility. As a result, postprocessing is strongly advised for 3D printing manufacturing [5,11,18].

In dental PMMA studies, water sorption, water solubility and the diffusion coefficient are therefore analysed together: sorption and solubility quantify how much water is retained and how much material is lost, while diffusion explains how fast these processes occur, which is crucial for predicting dimensional changes, surface degradation and long-term clinical performance of interim restorative materials.

Surface morphology may be related to the chemical activity of alcoholic solvents, which can impair the network structure by causing polymer swelling and extracting uncured or partially cured components. Wash solutions may alter the resin’s residual monomer content and network structure and could change the resin’s surface texture [19]. Surface topography and roughness are recognised to have a direct impact on dental materials’ accuracy, water absorption, discolouration, and microbial adhesion [20,21].

Surface roughness may modulate liquid sorption dynamics and, consequently, influence the colour stability of resin materials [22].

The purpose of this study is to investigate how the fabrication method (milling vs. 3D printing) influences the water sorption and solubility capacity over time, and how the surface characteristics of digitally obtained PMMA dental materials govern their hydrolytic stability.

Null hypotheses were formulated as follows:

**H01:**  *Fabrication method has no significant effect on water sorption and solubility.*

**H02:**  *Water absorption kinetic profiles (at different points in time) do not differ significantly between the two fabrication methods.*

**H03:**  *There is no interaction between fabrication method and immersion time on water sorption.*

**H04:**  *Surface roughness does not differ significantly between milled and 3D-printed PMMA.*

**H05:**  *Surface topography does not significantly predict hydrolytic degradation in PMMA dental materials.*

## 2. Materials and Method

### 2.1. Specimen Preparation

For the current study, 56 (*n* = 8) samples of digitally processed materials (Table 1), divided into seven groups, were prepared in the shape of a parallelepiped with dimensions of 10 mm × 10 mm × 1 mm. Groups (A) I–III of experimental samples were achieved from CAD/CAM resin discs for subtractive manufacturing. Groups (B) IV–VII of experimental samples were made using the 3D printing method, with different postprocessing methods. Using Autodesk Fusion software (Autodesk Ink, San Francisco, CA, USA, SUA)—version 2.0.21550, the samples were created as 10 mm × 10 mm × 1 mm plates and exported as stereolithographic (.stl) files. A Digital Light Processing (DLP) printer, Asiga UV Max (Asiga, Sydney, NSW, Australia), with a wavelength of 385 nm and a layer thickness of 50 μm, had its printing parameters set in accordance with the manufacturer’s instructions. To remove any residual uncured resin, the samples were cleaned with pure isopropyl alcohol for 5 min. After postprocessing procedures, the specimens were divided into four groups. Post-curing equipment BB Midi Plus (MECCATRONICORE, Pergine Valsugana, Trentino, Italy), with triple-frequency technology (365, 405, 480 nm), was used to perform the post-curing procedure. Specimens were polished up to grit P2000.

### 2.2. Water Sorption (wsp) and Water Solubility (wsl) Protocol and Water Diffusion Coefficient (D)

The prepared specimens (10 × 10 × 1 mm) (*n* = 56) were moved to a desiccator with silica on the base in order to speed up the drying process. The desiccator was kept at 37 °C in an incubator. Next, each specimen’s weight was determined using analytical balance AS 220.R2 PLUS (Synergy Lab line-RADWAG, Radom, Mazowieckie, Poland). This process was repeated until each specimen’s mass loss did not exceed 0.1 mg over 24 h. The first weighing was performed and recorded as M1, denoting the initial dry mass after storage in a desiccator at 37 °C, when the specimens’ mass loss fell below 0.1 mg. Digital callipers were used to determine each specimen’s volume based on its geometric specifications. After being fully submerged in distilled water, the specimens were incubated at 37 °C for 24 h, 48 h, 72 h, 7 days and 14 days. Following removal, absorbent paper was used to gently blot the specimens until no discernible moisture remained on the surface. For each step, the mass was measured immediately and recorded as M2, denoting the mass after water immersion. Specimens were again placed in a desiccator at 37 °C for a further 24 h to eliminate any remaining moisture. Subsequently, the final weighing was performed and recorded as M3, which corresponded to the final dry mass. According to ISO 10477:2018 [23], which defines M1 as the initial dry mass, M2 as the wet mass after immersion, and M3 as the final dry mass after re-drying, these three values (M1, M2, and M3) were used to compute water sorption (wsp) and water solubility (wsl). The following formulas were used to determine the wsp and wsl values.

Equations (1) and (2) are used to represent the water sorption, wsp, and water solubility, wsl, in µg/mm^3^:

wsp = (M2 − M3)/V (µg/mm^3^) (1)

wsl = (M1 − M3)/V (µg/mm^3^) (2)

where M1 stands for constant mass dry samples, M2 for constant mass wet samples, and M3 for constant mass reconditioned samples.

The Fickian diffusivity (D) of a dental PMMA can be estimated directly from the half-time t_1/2_ and the specimen thickness L.

D = 0.049 × L^2^/t_1/2_ (3)

### 2.3. AFM Nanoroughness Measurements and Topographic Analyses

An atomic force microscope (NanosurfEasyScan 2 Advanced Research, Nanosurf AG, Liestal, Switzerland) operating in contact mode was used to evaluate samples from each group. The AFM height data were utilised to compute the average nanoroughness Sa (nm) and the maximum height amplitude Sy (nm). Three-dimensional topographic images of the specimen surfaces were produced over scan regions of 2.2 μm × 2.22 μm. AFM allows quantitative measurement of surface topography beyond the optical diffraction limit by using a sharp tip to examine the surface with lateral resolution in the nanometer range and sub-nanometer vertical sensitivity. It allows surface property evaluations.

### 2.4. Statistical Analyses

Two statistical software packages were employed for data analysis: JASP (JASP Team, University of Amsterdam, The Netherlands, version 0.16.2) and IBM SPSS Statistics (IBM Corp., Armonk, NY, USA, version 31.0). Descriptive statistics were initially calculated to provide an overview of data distribution and central tendencies. For normally distributed variables, means and standard deviations (SD) were reported. For non-normally distributed data, medians and interquartile ranges (IQR) were calculated. The normality of the data distribution was assessed using the Shapiro–Wilk test. Because the assumption of normality was not met, non-parametric statistical tests were subsequently applied. The Mann–Whitney U test was used to compare two independent groups within the same experimental stage. Intragroup comparisons of materials (subtractive or additive) within a specific stage were performed using the Kruskal–Wallis test, followed by a Dunn-type post-hoc test for multiple pairwise comparisons. Differences between two time points (distinct experimental stages) were evaluated using the Friedman test. Spearman’s rank-order correlation analysis was conducted to assess the relationships between wsp, wsl, Sa, and Sy parameters. The coefficient of determination (r^2^) was calculated to evaluate predictive strength and to identify significant predictors. The level of statistical significance was set at α = 0.05.

## 3. Results

The study evaluated PMMA samples—milled PMMA (A: I, II, III) and 3D-printed PMMA (B: IV, V, VI, VII)—under different immersion durations: 24 h, 48 h, 72 h, 7 days, and 14 days.

For variables that did not meet the assumption of normal distribution, measures of central tendency and dispersion were expressed as the median (reflecting the 50th percentile) and the interquartile range (IQR), defined as the interval between the 25th and 75th percentiles, in order to provide a robust and distribution-independent summary of the data (Table 2).

**Figure 1:**
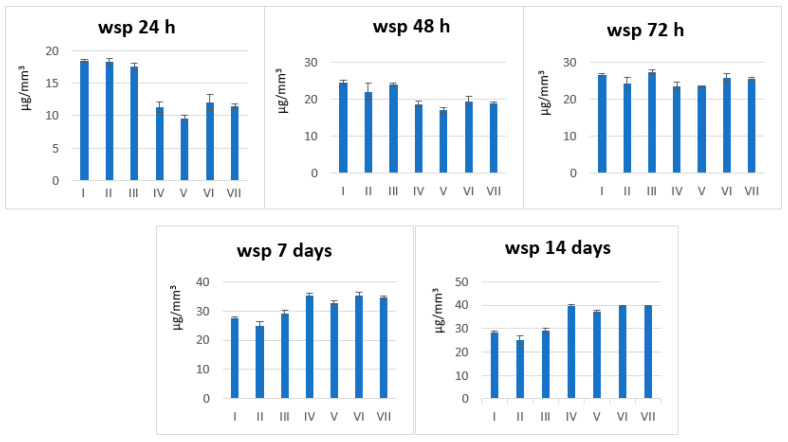
Average wsp values ± SD for all materials (I–VII) at different points in time: 24 h, 48 h, 72 h, 7 days, and 14 days.

**Figure 2:**
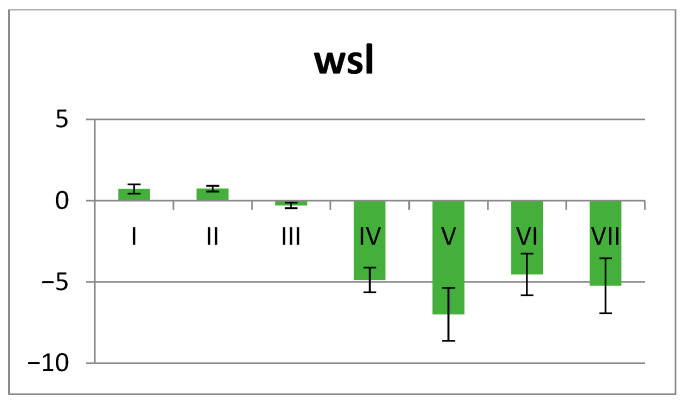
Average wsl values ± SD for all materials (I–VII).

Average (AVG) and standard deviation (SD) of wsp and wsl for each group/time were calculated. SD values generally increase over time in printed groups, indicating greater sample inconsistency in water uptake compared to milled ones (Figure 1 and Figure 2).

Milled PMMA shows solubility. Mass loss indicates true material dissolution, with soluble components leaching from polymer, and dimensional changes likely occurring.

3D-printed PMMA shows water retention. Mass gain indicates water incorporation, water becomes bound within the polymer matrix, and swelling and plasticisation likely occur.

**Figure 3:**
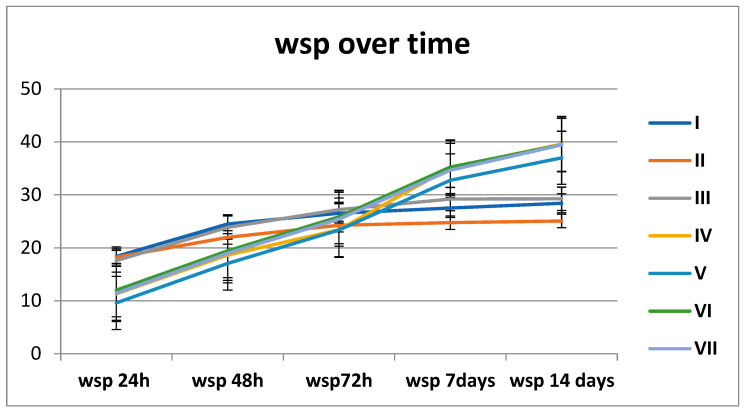
Average wsp values for all materials (I–VII) over time (24 h, 48 h, 72 h, 7 days, 14 days).

Milled groups (A: I–III) show a gradual increase in absorption over time, stabilising after ~7 days. Printed groups (B: IV–VII) start lower at 24 h but exhibit a sharp rise after 72 h, exceeding milled groups by 14 days. Printed materials show biphasic behaviour: initial resistance followed by accelerated sorption. Milled materials show monophasic behaviour: a gradual approach to equilibrium (Figure 3).

Printed PMMA absorbs more water and retains more after drying. Milled PMMA absorbs less water and loses material after drying.

These materials respond differently to prolonged water immersion. While milled PMMA exhibits a gradual, near-linear absorption profile, printed PMMA demonstrates a biphasic absorption pattern with accelerated uptake between 72 h and 7 days.

Following the application of the Shapiro–Wilk test, it was determined that the data were not normally distributed (*p* < 0.05), and consequently, non-parametric tests were performed.

The Mann–Whitney U test was used to compare two different groups (milled materials with additive ones) within an experimental stage (24 h, 48 h, etc), and significant differences (*p* < 0.001—for all studied time points) were found between all groups. The effect size r was calculated for Mann–Whitney U (r=1; 0.9; 0.6; −1; −1; 1, for 24 h, 48 h, 72 h, 7 days and 14 days).

**Figure 4:**
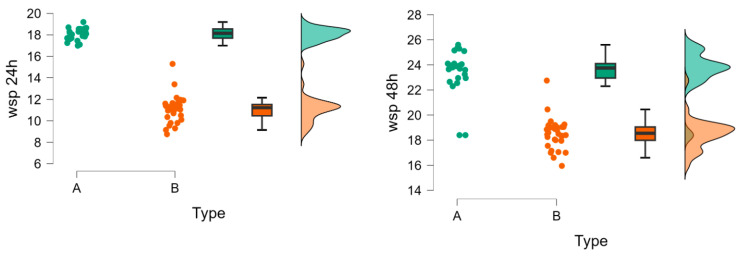

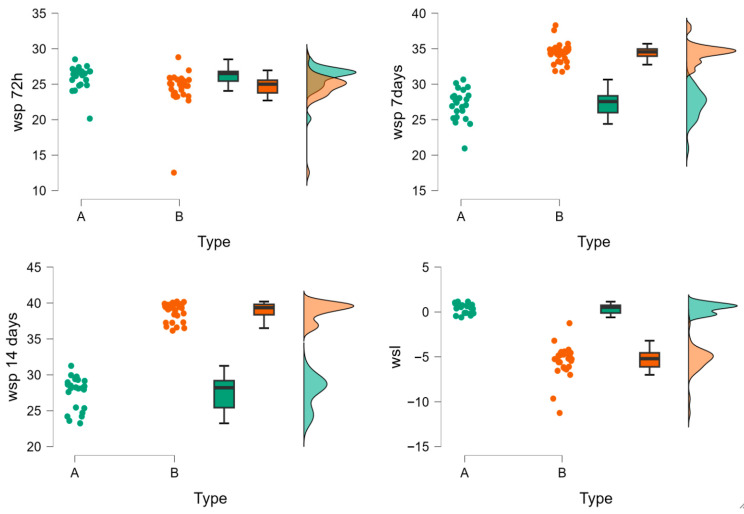
Raincloud plots for wsp and wsl over the analyses period. A = milled group (green), B = additive processed group (red).

The wsp additive processed samples (B) show much lower water sorption than the milled samples (A) at 24 h. Water sorption remains lower for type B at 48 h and 72 h. After 7 days, the trend reverses, and wsp becomes higher for type B (and lower for type A). At 14 days, the same pattern as at 7 days is maintained. For wsl type B, water solubility is significantly lower (Figure 4).

To compare the materials within a group (subtractive or additive), in a stage, the Kruskal–Wallis test and Tukey post hoc test (for multiple comparisons of the means) were applied.

**Figure 5:**
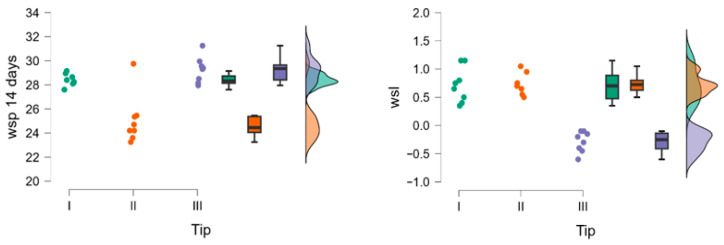
Raincloud plots for wsp and wsl after 14 days for group A. Material (I-red, II-green, III-blue).

Within group A, at 24 h, material III shows the lowest values. The Kruskal–Wallis test indicates significant differences among the three materials, and the post hoc pairwise comparisons show that there are no differences between I and II. After 48 h, material II shows the lowest values. The Kruskal–Wallis test indicates highly significant differences, while there are no differences between I and III. At 72 h, the Kruskal–Wallis test shows significant differences among the three materials, but in the post hoc pairwise comparison, there are no differences between I and III. At 7 days significant differences among the three materials are registered. At 14 days, the Kruskal–Wallis test shows highly significant differences, while the post hoc analysis indicates non-significant differences between I and III. Regarding wsl material, III shows the lowest values. The Kruskal–Wallis test reveals significant differences among the three materials, while the post hoc pairwise comparison indicates no differences between I and II (Figure 5).

**Figure 6:**
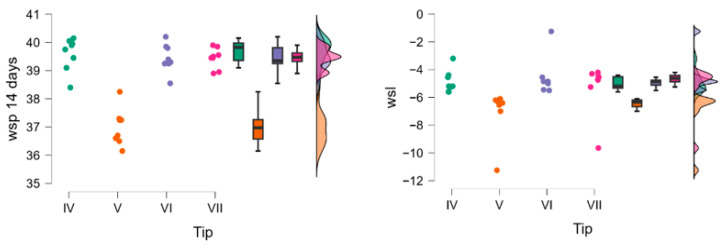
Raincloud plots for wsp and wsl after 14 days for group B. Material (IV-red, V-green, VI-blue, VII-pink).

For group B, the Kruskal–Wallis test shows significant differences at 24 h; however, in the pairwise comparisons, there are no differences between IV and VI or between VI and VII. At 48 h, the Kruskal–Wallis test shows differences, but in the pairwise comparisons, there are no differences between IV and VI, IV and VII, or VI and VII. At 72 h, there are no significant differences. At 7 days, the Kruskal–Wallis test shows differences among the four materials, but the post hoc analysis indicates no differences between IV and VI, IV and VII, or VI and VII. At 14 days, the Kruskal–Wallis test shows differences, and the post hoc analysis indicates no differences between IV and VI, IV and VII, and VI and VII. For wsl, the Kruskal–Wallis test shows differences, but the post hoc analysis indicates no differences between IV and VI, IV and VII, V and VII, or VI and VII (Figure 6, Table 3).

**Figure 7:**
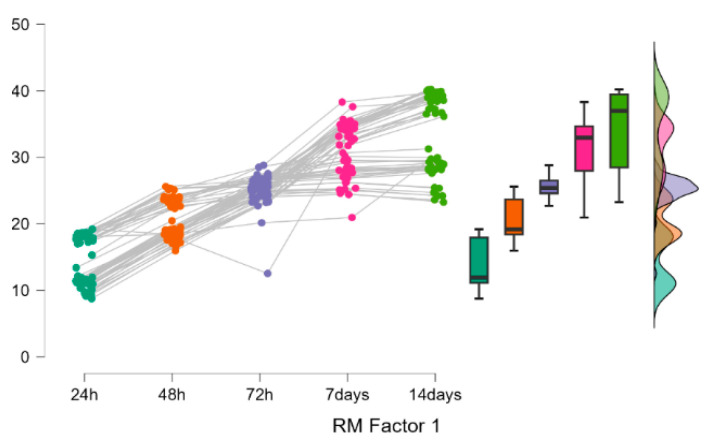
Raincloud plots for wsp group A compared to wsp group B during the analysis period. 24 h-dark green,48 h-red,72 h-blue, 7 days-pink, 14 days-light green.

To assess the temporal evolution over time of the two materials categories, the Friedman test (*p* < 0.001) and post hoc comparisons (*p* < 0.001) were applied. Materials belonging to both groups (A and B) were compared for two points (different stages), and the effect size was calculated for the Friedman tests $\eta^{2}=0.9$ (Figure 7, Table 4).

Diffusion coefficient was calculated and values as D = 1.02–1.15 × 10^−12^ m^2^/s for milled and 0.85 × 10^−12^ m^2^/s for printed materials were obtained.

### Nanoroughness and Topography by AFM

**Figure 8:**
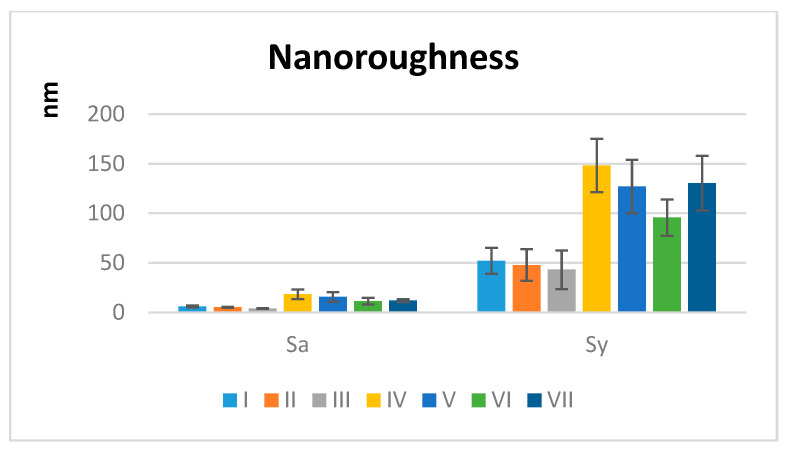
Average nanoroughness values ± SD for all materials (I–VII): average nanoroughness Sa (nm) and the maximum height amplitude Sy (nm).

The measured nanoroughness data strongly support the hypothesis that the fabrication method indicates nanoscale surface topography. Groups IV–VII have a drastically higher actual surface area exposed to water compared to their projected area. This may accelerate water sorption, promoting the hydrolytic degradation of the polymer matrix (Figure 8).

**Figure 9:**
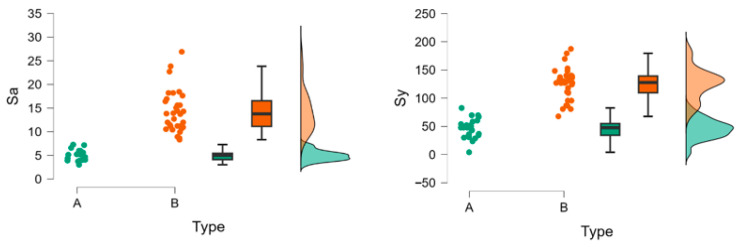
Raincloud plots for nanoroughness for groups A and B. A = milled group (green), B = additive processed group (red).

The Mann–Whitney test comparing Sa (Sy) in group A with Sa (Sy) in group B is highly significant (*p* < 0.01), and the values in group A are lower than those in group B (A < B) (Figure 9).

Spearman correlations were used to measure the association between two variables (wsp and Sa/Sy, wsl and Sa/Sy).

r = −0.3 indicates an inverse correlation between wsp and Sa, of moderate strength, with *p* = 0.2, which is not statistically significant. r^2^ = 0.09 means that 9% of the variance in WSP is explained by Sa. Between wsp and Sy, r is close to 0, indicating no association between wsp and Sy, and the correlation is not statistically significant.

**Figure 10:**
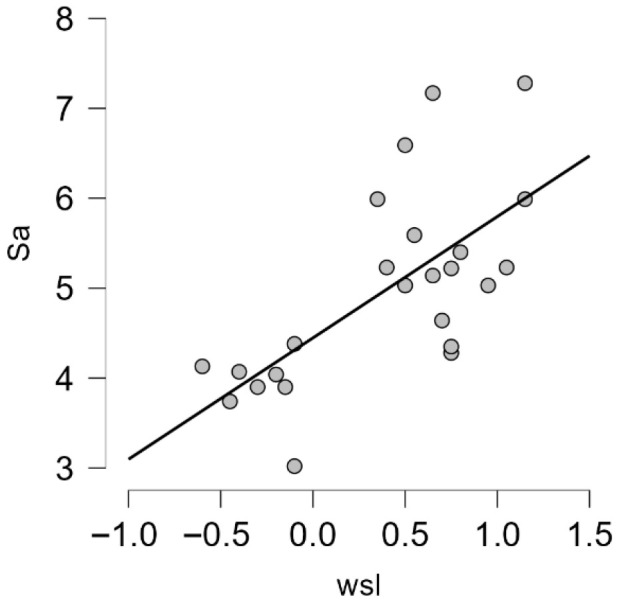
Graphic representation of the strong positive correlation between Sa and wsl.

For wsl and Sa, r = 0.64 indicates a strong positive correlation. r^2^ = 0.409 means that 40% of the variance is explained (a large effect), and *p* < 0.001 is highly significant, so the result can be generalised (Figure 10). For wsl and Sy, r = 0.1 indicates a direct, positive correlation, but it is weak in terms of strength. r^2^ = 0.01 means that 1% of the variance is explained, and since *p* > 0.005, the result is not statistically significant.

**Figure 11:**
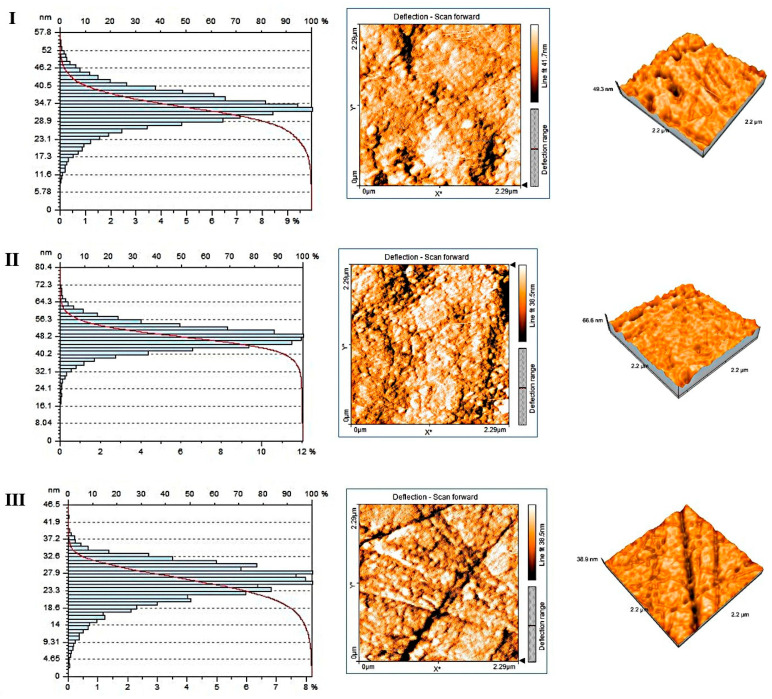
AFM 3D topography maps, 2D height (colour) maps, and corresponding height-distribution histograms of milled PMMA surfaces (for group A-Material I, II and III).

The images represent AFM 3D topography maps, 2D height (colour) maps, and corresponding height-distribution histograms of polished dental PMMA surfaces, recorded on three milled PMMA materials (first three panels) and four 3D-printed PMMA materials (last four panels). The 3D views show the vertical relief and defect morphology, the colour maps encode local height, and the histograms quantify how the surface heights are distributed across the scanned area, allowing a visual assessment of roughness, homogeneity, and the presence of peaks/valleys at the nanoscale (Figure 11).

**Figure 12:**
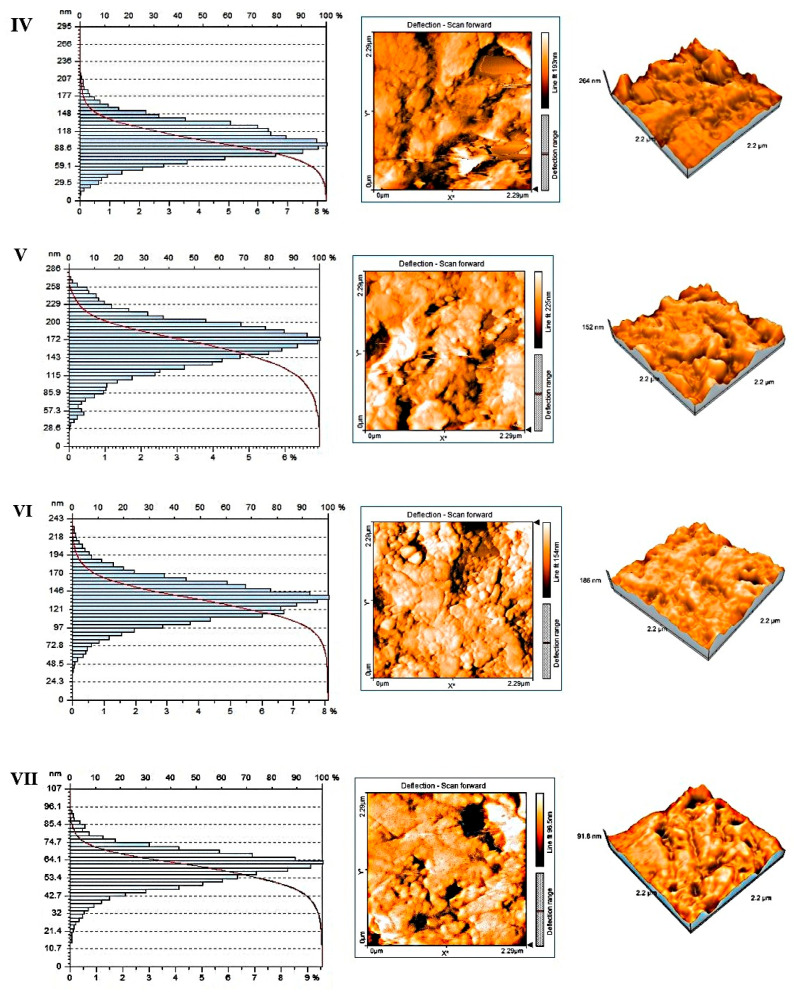
AFM 3D topography maps, 2D height (colour) maps, and corresponding height-distribution histograms of printed PMMA surfaces (for group B-Material IV, V, VI, and VII).

The 3D AFM images and colour maps of the milled PMMA specimens (I–III) usually show comparatively smooth, gently undulating surfaces without noticeable pits or sharp spikes, which is consistent with industrially polymerised, high-density PMMA that has been polished to a uniform finish. Height-distribution histograms indicate a more isotropic, defect-poor morphology and lower Sa/Sq values, indicating that the majority of surface points lie close to the mean plane with only slight deviation (Figure 12).

According to the literature, this morphology is consistent with CAD/CAM PMMA blocks that have minimal internal porosity and a high degree of conversion, in which polishing reveals a dense, continuous PMMA matrix and eliminates machining marks. Because there are fewer nanoscale locations for water infiltration, plasticisation, or leaching, these surfaces often have lower water sorption and solubility and higher mechanical stability.

For the 3D-printed PMMA specimens (IV–VII), the AFM 3D views and colour maps usually show more pronounced local height variations, with distinct ridges, depressions, or micro-steps that most likely correspond to layer lines, interlayer boundaries, or partially filled voxels intrinsic to the additive manufacturing process. With longer tails toward higher peaks or deeper valleys, height-distribution histograms are wider and occasionally asymmetrical, indicating greater deviations from the mean plane and a higher density of nanoscale defects.

This topography is typical of photopolymer 3D-printed dental resins, where anisotropic roughness and nanoporosity are produced by layer-wise construction and potential under-polymerisation. The higher solubility often reported for printed interim materials in comparison to milled PMMA can be explained by these nanoscale features, which increase the effective surface area and create capillary and diffusion pathways that can favour water uptake and localised accumulation. They may also facilitate the elution of residual monomers or additives from near-surface regions.

## 4. Discussion

Regarding the relatively new technology of CAM processing, it has been demonstrated that 3D additively created resin-based materials are more prone to water sorption than their milled, or conventional, counterparts [4,7,24,25,26].

**Figure 13:**
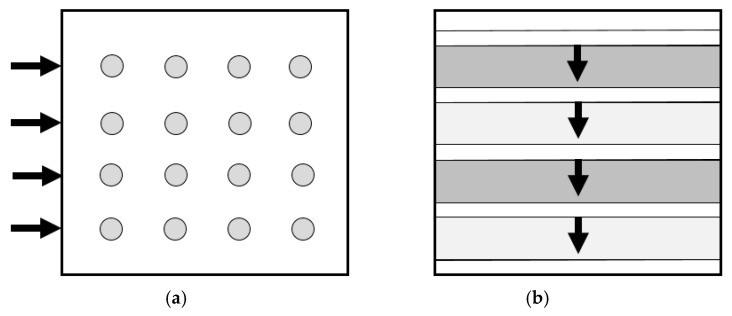
Schematic comparison of microstructure and water diffusion pathways (arrow directions) in milled (**a**) vs. additively (**b**) manufactured PMMA.

Water sorption is one of the characteristics of acrylic resins. This affinity for water modifies the resin’s physical characteristics and causes dimensional changes that lead to internal tensions, which negatively affect clinical performance. Water solubility is an undesired feature [4,27,28]. To avoid fractures and cracks, it is crucial to make sure that the material’s water sorption/solubility qualities are kept to a minimum. The capacity of dental materials to absorb liquids and alter their weight and volume is known as water sorption. This process is both chemical and physical [3]. The schematic visualisation of the microstructure and water diffusion pathways in milled PMMA (homogeneous network—uniform Fickian diffusion) and additively manufactured PMMA (layered structure—preferential interfacial diffusion) is presented in Figure 13. A higher diffusion coefficient D means water enters the PMMA faster, resulting in early changes in hardness and flexural strength.

When producing 3D-printed resin prosthesis, water absorption and solubility are crucial considerations. Long-term water absorption by the resin may cause internal stress and fissures, which could adversely affect the prosthesis’s characteristics [16].

Milled resins exhibited the lowest surface nanorougness, the highest microhardness and elastic modulus compared to printed ones [29]. A significant positive correlation between flexural strength and microhardness was found for subtractively and additively manufactured PMMA [30]. It is assumed that hardness is influenced by the residual monomer content, its values being directly proportional to the amount of residual monomer present in the material [31,32,33]. The chemical composition of additively manufactured materials varies significantly, and the details of the production process for pre-polymerised blocks for CAD/CAM technology are trade secrets; they can be obtained through different industrial procedures, making the comparison of various studies potentially difficult [34,35]. A possible conclusion would be that certain mechanical and surface properties depend on the choice of material and not on the manufacturing process [30].

Research revealed that the printed layering method might be the reason for the enhanced water sorption. Absorbed water fills the voids and interpolymeric spaces between the resin’s polymer layers, displacing the polymer chains from one another. Changes in mechanical strength, slight chemical degradation, and the elution of residual monomers can result from interactions between water and polymer chains. Free-radical polymerisation can alter the density of methacrylate-based resin composites, resulting in heterogeneity in the polymerised material that may make it easier to trap leftover monomers and remove them [3,36]

The filler content, the hydrophilic or hydrophobic nature of the resin matrix, and the filler–matrix interface—which may serve as a site for additional water retention, particularly if the bond at this interface is weak—all have an impact on the water sorption and solubility of resin materials [37].

Industrial CAD/CAM PMMA blocks have a very high degree of conversion (DC) compared with conventional or 3D-printed resins, but DC is not given as a numeric value by the manufacturers and must be deduced from residual-monomer and property data. Scientifically, their high DC comes from high-temperature, high-pressure industrial polymerisation, which reduces free monomer, porosity, and water sorption/solubility and increases flexural strength and long-term stability [37].

The fabrication method is the primary determinant of hydrolytic stability in digitally processed PMMA. Therefore, the null hypothesis H01 (Table 5) is rejected. Milled PMMA (Group A) demonstrated a significantly lower water sorption and, critically, exhibited negative solubility (mass loss), indicative of true material dissolution. In contrast, 3D-printed PMMA (Group B) exhibited a significantly higher water sorption and positive solubility (mass gain), signifying water incorporation and swelling of the polymer matrix. This fundamental difference in mechanism confirms that manufacturing technology influences the core material’s response to a wet environment.

3D-printed and milled PMMA exhibit distinct and statistically different water absorption kinetics. The null hypothesis H02 (Table 5) is rejected.

Milled PMMA followed a monophasic, gradual absorption profile, approaching saturation within 7 days. Conversely, 3D-printed PMMA displayed a biphasic absorption pattern, characterised by a delayed, sharp increase in water uptake between 72 h and 14 days. This kinetic disparity suggests distinct underlying absorption mechanisms, most likely related to the material’s microstructure. The data reveal a significant interaction between fabrication method and immersion time; therefore, the null hypothesis H03 (Table 5) is rejected.

This hydrolytic stability is further influenced by the integrity of the filler–polymer interface, since weakened covalent bonds produce micro-voids that allow water molecules to infiltrate and cause polymer chain hydrolysis, dimensional instability, and accelerated leaching of unreacted components. These degradative processes collectively compromise mechanical integrity and functional longevity, requiring compliance with ISO 10477:2020 standards (wsp ≤ 40 μg/mm^3^, wsl ≤ 7.5 μg/mm^3^ after 7 days) to ensure adequate clinical performance [7,27,28,38].

Different photoinitiator systems significantly affect dental composites’ properties, with trimethylbenzoyldiphenylphosphine oxide (TPO)-based systems often showing superior colour stability compared to traditional camphorquinone (CQ) or bisacylphosphine oxide (BAPO), while conversion, water sorption, and solubility vary, with TPO-based materials sometimes meeting or exceeding clinical standards for colour stability (ΔE < 3.3) after ageing, though some studies suggest that the photoinitiator type does not always impact water sorption/solubility much [12,39].

The chemical components of the wash solutions also affected water sorption (wsp) and water solubility (wsl). Another characteristic affected by the washing was surface roughness [19]. The surface quality of resins may be compromised by thermal stressors and water exposure, potentially leading to water absorption. This could result in a reduction in the surface characteristics of the objects [27]. Therefore, the correlation with surface topography is important.

Prior studies have shown that extending the post-rinsing period adversely affects the surface topography of a 3D-printed resin. In particular, surface fissures were seen when the rinse period was extended to 12 h. The findings showed that although the surface morphology of a DLP-printed orthodontic appliance material was affected by several rinse solvents, the roughness values (Ra and Rz) were unaffected. The surface morphology of each specimen revealed a groove structure that was more or less pronounced [20,40,41]. In order to effectively maintain the mechanical and surface characteristics of these cutting-edge materials, future research should focus on optimising washing procedures [19,40].

For example, post-curing optimisation is crucial because it increases the cross-linking of unreacted chemical groups, especially when long-term photo and thermal polymerisation are combined. In order to reduce the amount of leftover monomer, processing enhancements concentrate on achieving a higher degree of double-bond conversion and ensuring full polymerisation [28,42].

The water sorption mechanisms of milled PMMA can be related to surface sorption (limited to superficial features), capillary action (regular grooves provide directional transport), bulk diffusion (slow penetration through dense polymer matrix), and saturation (reaching equilibrium by 7 days). For 3D-printed PMMA, the principles of water sorption are linked to surface wetting delay (initial hydrophobic response), capillary network activation (interconnected pores become accessible), interlayer diffusion (water penetrates between printed layers), and continued absorption (the porosity network allows extended uptake beyond 14 days).

Postprocessing parameters within 3D printing significantly modulate, but do not eliminate, material limitations. Among the 3D-printed groups, variations in post-curing time and temperature (e.g., Group V: 7 min at 60 °C; Group VI: 20 min at 22 °C) resulted in measurable differences in water sorption and solubility.

Surface properties are greatly influenced by material composition; methacrylate-based formulations often show better surface smoothness than bis-acryl alternatives because of improved filler–matrix integration and more predictable polymerisation kinetics [28]. The shortest curing time and the lowest oxygen concentration resulted in the least amount of water absorption [16].

Milled PMMA has smoother, more homogeneous AFM topography, and compact height distributions suggest a dense, well-polymerised surface with limited nanoscale defects, which is consistent with the low water sorption/solubility and good long-term stability reported for CAD/CAM PMMA blocks in the literature.

3D-printed PMMA shows more heterogeneous topography, with higher peaks and deeper valleys, and broader height distributions, indicating increased roughness and nanoporosity, which correlate with the higher solubility and more pronounced water-related degradation and monomer release observed for printed interim materials in recent studies.

AFM confirms a nanoscale morphological advantage of milled PMMA over 3D-printed PMMA, and that this difference provides a structural explanation for the lower water sorption and especially lower solubility measured in the milled group.

The quantitative AFM analysis provides statistically robust evidence (*p* < 0.001 for all major correlations) that surface roughness parameters (Sa, Sy) are primary determinants of water absorption behaviour in PMMA dental materials.

Additive manufacturing yields inherently higher nanoscale surface roughness than subtractive milling. The null hypothesis H04 (Table 5) is rejected. Atomic Force Microscopy (AFM) quantification confirmed a statistically significant and clinically relevant difference in surface topography. The 3D-printed groups (IV–VII) formed a distinct high-roughness cluster with average Sa values (11.18–18.20 nm) that were 2 to 4.7 times greater than the low-roughness cluster of milled groups (I–III) with Sa values of 3.90–6.00 nm. This nanoscale roughness is a direct consequence of the layer-by-layer fabrication process.

The three milled PMMA materials show significantly smoother, more homogeneous surfaces and lower, slower water sorption compared with the four 3D-printed PMMA materials, which exhibit markedly higher nanoscale roughness and faster, higher water uptake approaching ISO limits. This pattern fits the known differences between industrially polymerised CAD/CAM PMMA blocks and layer-wise printed methacrylate resins in terms of degree of conversion, porosity and long-term stability.

Surface topography is not a strong, quantitative predictor of hydrolytic degradation in PMMA. The null hypothesis H05 (Table 5) is partially accepted. A strong positive correlation was observed only between Sa and water solubility capacity. This provides a mechanistic link between the fabrication-induced surface characteristic and the materials’ performance. The increased effective surface area and the presence of nanoscale peaks and valleys in 3D-printed materials create capillary pathways and sites for water entrapment, which explains their water uptake.

This study has several inherent limitations. First, the experiments were conducted under controlled laboratory conditions, as a simplified model of the in vivo state, and do not fully replicate the complex, dynamic oral environment with its thermal fluctuations, pH changes, and microbial activity, as samples were immersed in distilled water. Second, the investigation was limited to a small number of resin systems; therefore, the findings may not be directly extrapolated to other brands or formulations with differing polymer chemistry. Third, the evaluation time (14 days) provides insights into short-term hydrolytic kinetics, but may not capture very long-term degradation or saturation plateau effects. Finally, the study focused on physical sorption properties without correlating them with critical mechanical properties, which are essential for predicting clinical performance.

To fully benefit from digital manufacturing in prosthetics, it is recommended that additive resins be improved. Targeted research should investigate the composition (addition of nanoparticles and nanocomposites) and the optimisation of printing parameters—such as number and layer thickness, exposure time, and build orientation—in order to minimise surface roughness while maintaining dimensional accuracy. Developing and standardising effective postprocessing protocols, including chemical polishing or novel coating techniques, is a promising avenue for improving the surface integrity of 3D-printed PMMA.

As a clinical recommendation, milled PMMA remains preferable for long-term applications requiring dimensional stability, whereas 3D-printed PMMA may be suitable for short-term use, with appropriate monitoring and, if necessary, surface treatments to mitigate its higher absorption.

## 5. Conclusions

This study indicates that milled PMMA, derived from industrially polymerised blocks, offers superior hydrolytic stability, characterised by lower water sorption, true solubility (leaching), and a smooth, defect-poor surface. 3D-printed PMMA, while offering geometric freedom, presents a distinct risk profile defined by higher equilibrium water uptake, water-induced swelling, and a rough, nanoporous surface that actively promotes these processes. Therefore, material selection must be application-specific: milled PMMA remains the benchmark for long-term provisional restorations requiring maximum durability and dimensional stability, whereas 3D-printed PMMA may be suitable for short-term applications where its sorption kinetics and surface-driven degradation can be clinically managed.

## Figures and Tables

**Table 1:** Materials and fabrication methods investigated in the study.

Group (No.)	Material Shade	Manufacturer	Fabrication Method	Composition	Postprocessing
I	Telio CAD A2	IvoclarViva-dent AG, Liechtenstein	Cutting prom polymerised discs	PMMA (polymethyl methacrylate) with colour pigments	Standard mechanical polishing
II	Shaded PMMA A2	Dentsply Sirona, Bensheim, Germany	Cutting prom polymerised discs	PMMA (polymethyl methacrylate) with colour pigments	Standard mechanical polishing
III	Copra Temp Symphony A2	Whitepeaks Dental Solutions GmbH, Hamminkeln, Germany	Cutting prom polymerised discs	PMMA (polymethyl methacrylate) with colour pigments	Standard mechanical polishing
IV	Lumina/A2	Dentona AG, Dortmund, Germany	3D printing Layer thickness 50 µm, wavelength 385 nm	Methacrylate-based photopolymer with inorganic fillers and pigments and a photoinitiator system	Rinse in isopropanol ultrasonic bath 5 min Postcuring time 7 min, temperature 22 °C Standard mechanical polishing
V	Lumina/A2	Dentona AG, Dortmund, Germany	3D printing Layer thickness 50 µm, wavelength 385 nm	Methacrylate-based photopolymer with inorganic fillers and Pigments and a photoinitiator system	Rinse in isopropanol ultrasonic bath 5 min Postcuring time 7 min, temperature 60 °C Standard mechanical polishing
VI	Lumina/A2	Dentona AG, Dortmund, Germany	3D printing Layer thickness 50 µm, wavelength 385 nm	Methacrylate-based photopolymer with inorganic fillers and pigments and a photoinitiator system	Rinse in isopropanol ultrasonic bath 5 min Postcuring time 20 min, temperature 22 °C Standard mechanical polishing
VII	Lumina/A2	Dentona AG, Dortmund, Germany	3D printing Layer thickness 50 µm, wavelength 385 nm	Methacrylate-based photopolymer with inorganic fillers and pigments and a photoinitiator system	Rinse in isopropanol ultrasonic bath 5 min Postcuring time 7 min, temperature 22 °C, oxygen inhibition glycerin Standard mechanical polishing

I, II, III—materials processed by subtraction; IV, V, VI, VII—materials processed by addition.

**Table 2:** Median and IQR for non-parametric data.

		I	II	III	IV	V	VI	VII
wsp 24 h	median	18.4	18.2	17.6	11.1	9.7	11.6	11.4
	IQR	0.3	0.4	0.5	0.5	0.6	0.2	0.3
wsp 48 h	median	24.5	22.0	23.8	18.6	17	19.4	19
	IQR	1.2	1.7	0.3	0.5	0.4	0.6	0.2
wsp 72 h	median	26.5	24.8	27.0	24.7	23.4	25.5	25.4
	IQR	0.3	1.0	0.6	0.6	0.4	0.4	0.8
wsp 7 days	median	27.5	25.1	29.4	35	32.9	34.9	34.5
	IQR	0.9	1.0	1.4	0.7	0.9	0.5	0.4
wsp 14 days	median	28.3	24.4	29.4	39.8	37	39.3	39.5
	IQR	0.6	1.3	1.2	0.6	0.7	0.6	0.3
wsl	median	0.7	0.7	−0.3	−5.2	−6.3	−4.8	−4.6
	IQR	0.4	0.2	0.3	0.8	0.5	0.3	0.5

median and IQR = measures of central tendency and dispersion.

**Table 3:** The comparison of the materials in a group (subtractive or additive) at a stage.

A	24 h	48 h	72 h	7 Days	14 Days
pKr.-Wall.	1.8 × 10^−2^	3.9 × 10^−3^	<0.001	<0.001	4.3 × 10^−3^
*p*-Dunn	I–II: 0.5	I–II: <0.001	I–II: 1.2 × 10^−3^	I–II: 1.8 × 10^−2^	I–II: 4.2 × 10^−2^
	I–II: 6.3 × 10^−3^	I–II: 0.1	I–III: 7.7 × 10^−2^	I–III: 4 × 10^−2^	I–III: 0.2
	II–III: 4 × 10^−2^	II–III: 8.6 × 10^−2^	II–III: <0.001	II–III: <0.001	II–III: 1.1 × 10^−3^
**B**					
pKr.-Wall.	<0.001	<0.001	<0.001	<0.001	<0.001
*p*-Dunn	IV–V: 1 × 10^−2^	IV–V: 1.6 × 10^−2^	IV–V: 7.4 × 10^−2^	IV-V: <0.001	IV–V: <0.001
	IV–VI: 7.6 × 10^−2^	IV–VI: 0.1	IV–VI: 3.6 × 10^−2^	IV–VI: 0.9	IV–VI: 0.5
	IV–VII: 0.5	IV–VII: 0.2	IV–VII: 5.3 × 10^−2^	IV–VII: 0.4	IV–VII: 0.5
	V–VI: <0.001	V–VI: <0.001	V–VI: <0.001	V–VI: <0.001	V–VI: 1.7 × 10^−3^
	V–VII: <0.001	V–VII: <0.001	V–VII: <0.001	V–VII: 6 × 10^−3^	V–VII: 1.3 × 10^−3^
	VI–VII: 0.3	VI–VII: 0.8	VI–VII: 0.9	VI–VII: 0.3	VI–VII: 0.9

A = group of materials processed by subtraction (I, II, III); B = group of materials processed by addition (IV, V, VI, VII).

**Table 4:** Post hoc comparisons—Type RM Factor 1.

		Mean Difference	SE	t	p_holm_
A, 24 h	B, 24 h	7.0	0.3	25.7	<0.001
A, 24 h	A, 48 h	−5.4	0.3	−20.6	<0.001
B, 24 h	B, 48 h	−7.4	0.2	−32.9	<0.001
A, 48 h	B, 48 h	4.9	0.4	12.2	<0.001
A, 48 h	A, 72 h	−2.6	0.4	−6.9	<0.001
B, 48 h	B, 72 h	−6.0	0.3	−18.7	<0.001
A, 72 h	B, 72 h	1.5	0.6	2.5	5.6 × 10^−2^
A, 72 h	A, 7 days	−1.2	0.4	−3.1	1.5 × 10^−2^
B, 72 h	B, 7 days	−9.9	0.3	−30.5	<0.001
A, 7 days	B, 7 days	−7.3	0.5	−15.3	<0.001
A, 7 days	A, 14 days	−0.4	0.3	−1.4	0.4
B, 7 days	B, 14 days	−4.4	0.3	−16.9	<0.001
A, 14 days	B, 14 days	−11.3	0.5	−24.1	<0.001

Note. *p*-value adjusted. A = group of materials processed by subtraction (I, II, III); B = group of materials processed by addition (IV, V, VI, VII).

**Table 5:** The description and status of each null hypothesis.

Null Hypotheses	Description of the Null Hypothesis	Status
H01	Fabrication method has no significant effect on the water sorption and solubility	Rejected
H02	Water absorption kinetic profiles (at different points in time) do not differ significantly between the two fabrication methods	Rejected
H03	There is no interaction between fabrication method and immersion time on water sorption	Rejected
H04	Surface roughness does not differ significantly between milled and 3D-printed PMMA	Rejected
H05	Surface topography does not significantly predict hydrolytic degradation in PMMA dental materials.	Partially accepted

H01, H02, H03, H04, H05—the null hypotheses in the study.

## Data Availability

The original contributions presented in this study are included in the article. Further inquiries can be directed to the corresponding author.
